# Temperature‐Modulated MEMS Gas Sensor Array Enabled by Physics‐Informed Deep Learning for Thermal‐Runaway Characteristic Gas Discrimination

**DOI:** 10.1002/advs.77784

**Published:** 2026-09-16

**Authors:** Qin Luo, Qi Guo, Rongyue Liu, Yilin Li, Haiping Zhu, Wei Zhang, Zhongren Chen, Zhaojun Liu, Xing Cheng

**Affiliations:** ^1^ College of Semiconductors (National Graduate College for Engineers) Southern University of Science and Technology Shenzhen China; ^2^ Department of Materials Science and Engineering Southern University of Science and Technology Shenzhen China; ^3^ Shenzhen Grubbs Institute and Department of Chemistry Southern University of Science and Technology Shenzhen China; ^4^ Department of Electrical and Electronic Engineering Southern University of Science and Technology Shenzhen China; ^5^ Advanced Materials Innovation Center Jiaxing Research Institute of Southern University of Science and Technology Jiaxing China

**Keywords:** deep learning, MEMS gas sensor array, temperature modulation, thermal runaway

## Abstract

Gas monitoring is a promising method for early warning of thermal runaway, yet existing sensor systems suffer from insufficient miniaturization, inadequate discrimination ability, and severe drift‐induced performance degradation. This work presents a 2 × 2 integrated single‐chip MEMS gas sensor array with temperature modulation and deep learning for discriminating thermal‐runaway characteristic gases. The array comprises four distinct temperature zones achieved by varying the line width of the heating electrodes, with the entire array measuring 1.8 × 1.8 × 0.5 mm^3^. Furthermore, it is capable of detecting four critical thermal‐runaway characteristic gases (H_2_, CO, CH_4_, and CO_2_) and exhibits distinct temperature‐modulated response patterns. To achieve reliable multi‐gas discrimination, we develop a Hierarchical Kolmogorov‐Arnold Mixture‐of‐Experts (HK‐MoE) model. Under single‐gas conditions, the model achieved initial F1‐scores of 99.53% for target gas presence detection, 99.88% for gas classification, and 98.09% for concentration determination. While performance experienced some degradation following a 30‐day continuous operational aging, the model consistently demonstrated satisfactory resilience and significantly outperformed conventional baselines and state‐of‐the‐art Transformer‐based time‐series models. Further validation using multi‐component gas mixtures yielded overall F1‐scores above 99.83% for all three tasks, demonstrating reliable mixed‐gas discrimination capability. This integrated framework offers a compact solution to drift‐induced instability, demonstrating its potential for reliable and early‐stage battery safety monitoring.

## Graphical abstract

This work presents a 2 × 2 integrated single‐chip MEMS gas sensor array with temperature modulation and deep learning for discriminating thermal‐runaway characteristic gases. The array generates distinct temperature‐modulated response patterns for four characteristic gases (H_2_, CO, CH_4_, and CO_2_), while a HK‐MoE model is developed to achieve robust discrimination under both stable and drifting conditions.

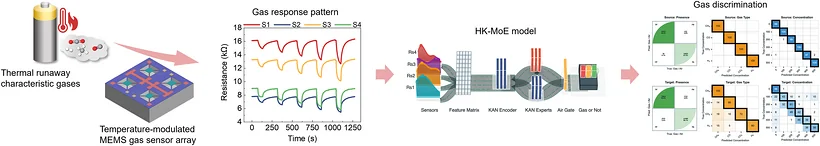

## 1 Introduction

With the large‐scale deployment of energy storage systems for power dispatch, renewable integration, and electric transportation, lithium‐ion batteries (LIBs) have become the dominant energy storage technology owing to their high energy density, high power capability, long cycle life, and relatively low environmental impact [1, 2, 3]. However, under abnormal conditions such as thermal, mechanical, or electrical abuse, LIBs are prone to thermal runaway, which can cause rapid temperature rise and may lead to fire or explosion [4, 5]. Such incidents not only incur equipment damage and economic loss but also pose significant threats to personnel safety. Therefore, timely monitoring and early warning of LIBs thermal runaway are of critical theoretical and practical importance.

Common methods for detecting thermal runaway include electrical, temperature, and pressure monitoring [6, 7]. However, external temperature and voltage sensors lack the sensitivity for early warning, while more advanced internal measurement techniques are hindered by high costs and integration complexities [8, 9, 10, 11]. Pressure sensors can detect small pressure changes but are prone to false alarms caused by temperature variations. In contrast, gas monitoring can detect thermal runaway up to 580 s earlier than traditional methods, making it the most promising approach for ultra‐early warning [12, 13]. Sophisticated analytical techniques, such as gas chromatography (GC) and mass spectrometry (MS), have identified H_2_, CO, CO_2_, and CH_4_ as the primary gases released during LIBs thermal runaway [14, 15, 16]. However, due to their bulky size and high cost, these instruments are confined to laboratory analysis.

Metal‐oxide‐semiconductor (MOS) gas sensors, particularly those fabricated using MEMS technology, have emerged as promising candidates for detecting early‐stage characteristic gases in LIBs thermal runaway due to their high sensitivity, rapid response, compact size, and low power consumption [17, 18, 19]. However, MEMS MOS gas sensors exhibit poor selectivity, rendering a single sensor inadequate for reliably distinguishing gas species during thermal runaway [20]. To address this limitation, gas sensor arrays combined with discrimination algorithms have been proposed [21, 22, 23, 24].

Most existing thermal‐runaway detection systems rely on arrays composed of multiple commercial MOS sensors fabricated with different sensing materials [25, 26]. Although material diversity can enhance intrinsic selectivity and thereby modestly improve recognition accuracy, this strategy introduces three fundamental limitations: (i) the increasing sensor count inevitably enlarges the overall system size and power consumption, hindering miniaturization; (ii) the distinct aging behaviors and drift characteristics among diverse materials cause varying baseline resistance shifts across sensing units, complicating model calibration and long‐term maintenance; and (iii) the traditional pattern‐recognition algorithms generally lack discrimination ability and drift robustness, restricting their reliability in real‐world deployment.

To address these challenges, temperature‐modulated sensor arrays have emerged as a compelling alternative by shifting the source of selectivity from material diversity to temperature‐dependent thermodynamic processes. Under dynamic temperature excitation, each gas can generate a unique response pattern because variations in operating temperature modulate the dominant surface‐adsorbed oxygen species, adsorption–desorption equilibria, and gas–solid reaction kinetics on the sensing layer [27, 28]. Consequently, there is a critical need to develop miniaturized, low‐power, single‐chip, temperature‐modulated gas sensor arrays integrated with advanced data‐processing algorithms to achieve reliable detection of thermal‐runaway characteristic gases.

In this work, we present a 2 × 2 single‐chip MEMS gas sensor array combined with a physics‐informed model for discriminating thermal‐runaway characteristic gases. The array measures 1.8 × 1.8 × 0.5 mm^3^ and comprises four sensor units with varying heating electrode line widths (8, 12, 16, and 20 µm). Under a constant heating voltage, four distinct temperature zones are generated, thereby achieving temperature modulation. Furthermore, it is capable of detecting four critical thermal‐runaway characteristic gases (H_2_, CO, CH_4_, and CO_2_) and exhibits distinct temperature‐modulated response patterns. The proposed model incorporates physics‐informed feature engineering and a Zero‐Gate mechanism for noise suppression, achieving highly reliable gas discrimination under single‐gas conditions, with F1‐scores of 99.53% for target gas presence detection, 99.88% for gas classification, and 98.09% for concentration determination. After 30 days of continuous operational aging, it maintained F1‐scores of 97.55%, 80.62%, and 57.68% for the three tasks, respectively, outperforming conventional baselines and state‐of‐the‐art Transformer‐based time‐series models. Although the model performance degraded noticeably after 60 days of aging, the proposed few‐shot calibration strategy effectively recovered the degraded performance using only a small number of labeled calibration samples, demonstrating its potential for long‐term deployment. Furthermore, additional validation on multi‐component gas mixtures demonstrated overall F1‐scores above 99.83% for all three tasks, highlighting excellent mixed‐gas discrimination capability. We believe that this integrated framework offers a compact and drift‐resilient solution to instability induced by long‑term sensor aging, holding significant potential for reliable, early‐stage battery safety monitoring.

## 2 Results and Discussions

### 2.1 Single‐Chip MEMS Gas Sensor Array

**FIGURE 1:**
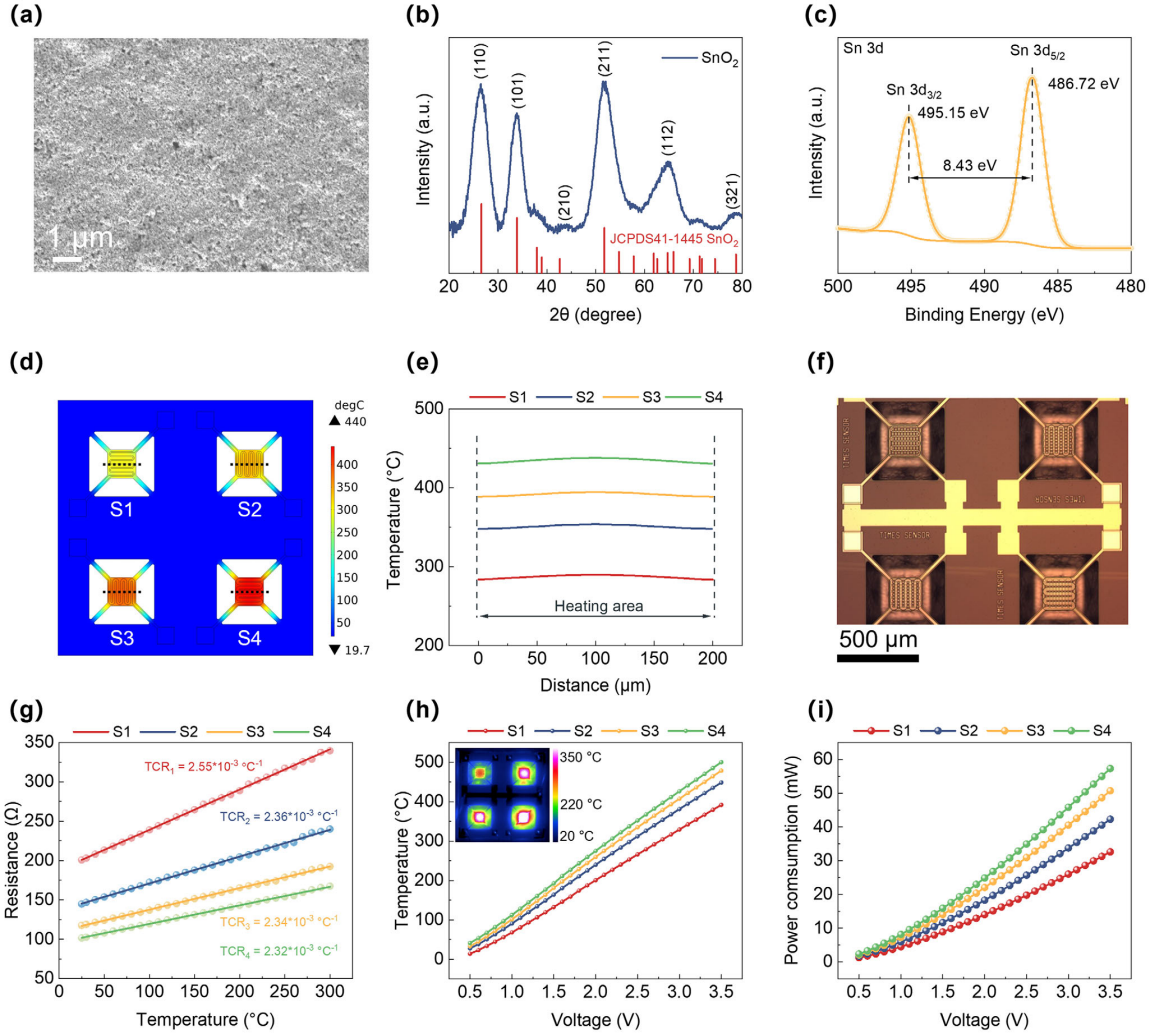
(a) SEM image of SnO_2_ nanoparticles, the inset shows the sensor array. (b) XRD pattern and (c) Sn 3d XPS spectrum of SnO_2_ nanoparticles. (d,e) COMSOL Multiphysics simulation results of the temperature fields in MEMS microheater array. (f) Optical microscope image of the fabricated MEMS microheater array chip. (g) The temperature–dependent resistance characteristics of the heating electrode. (h) The temperature versus applied heating voltage of the MEMS microheater array. (i) The power consumption versus applied heating voltage of the MEMS microheater array.

Figure 1a,b displays the XRD pattern and Sn 3d XPS spectrum of the SnO_2_ nanoparticles, respectively, confirming the successful synthesis of the material. Additionally, the nanoparticles demonstrate a uniform grain size distribution, as shown in Figure 1c. Figure 1d displays the COMSOL simulation of the single‐chip MEMS microheater array, showing uniform temperature distribution in each unit. Figure 1e plots the temperature along the horizontal centerline (dashed line in Figure 1d) at 3 V, revealing peak temperatures of 290°C, 354°C, 395°C, and 440°C, while maintaining temperature gradients below 1.24%. Figure 1f shows the optical microscope image of the array, featuring clear metal electrode contours, smooth edges, and no visible defects. Figure 1g depicts the temperature–dependent resistance characteristics of each unit, demonstrating a strong linear correlation. The observed decrease in temperature coefficients of resistance (TCR) with increasing Pt electrode width may be attributed to a longer and more uniform heat diffusion path in the resistance wire, resulting in a smaller resistance change. Figure 1h presents the voltage–dependent temperature characteristics and corresponding infrared thermal images of each unit. At 3 V, the units reach 329°C, 381°C, 407°C, and 426°C, respectively, showing good consistency with the COMSOL simulations. Figure 1i presents the voltage–dependent power consumption characteristics of each unit. At 3 V, the units consume 26, 34, 41, and 46 mW, respectively, resulting in a total power consumption of 147 mW for the array.

**FIGURE 2:**
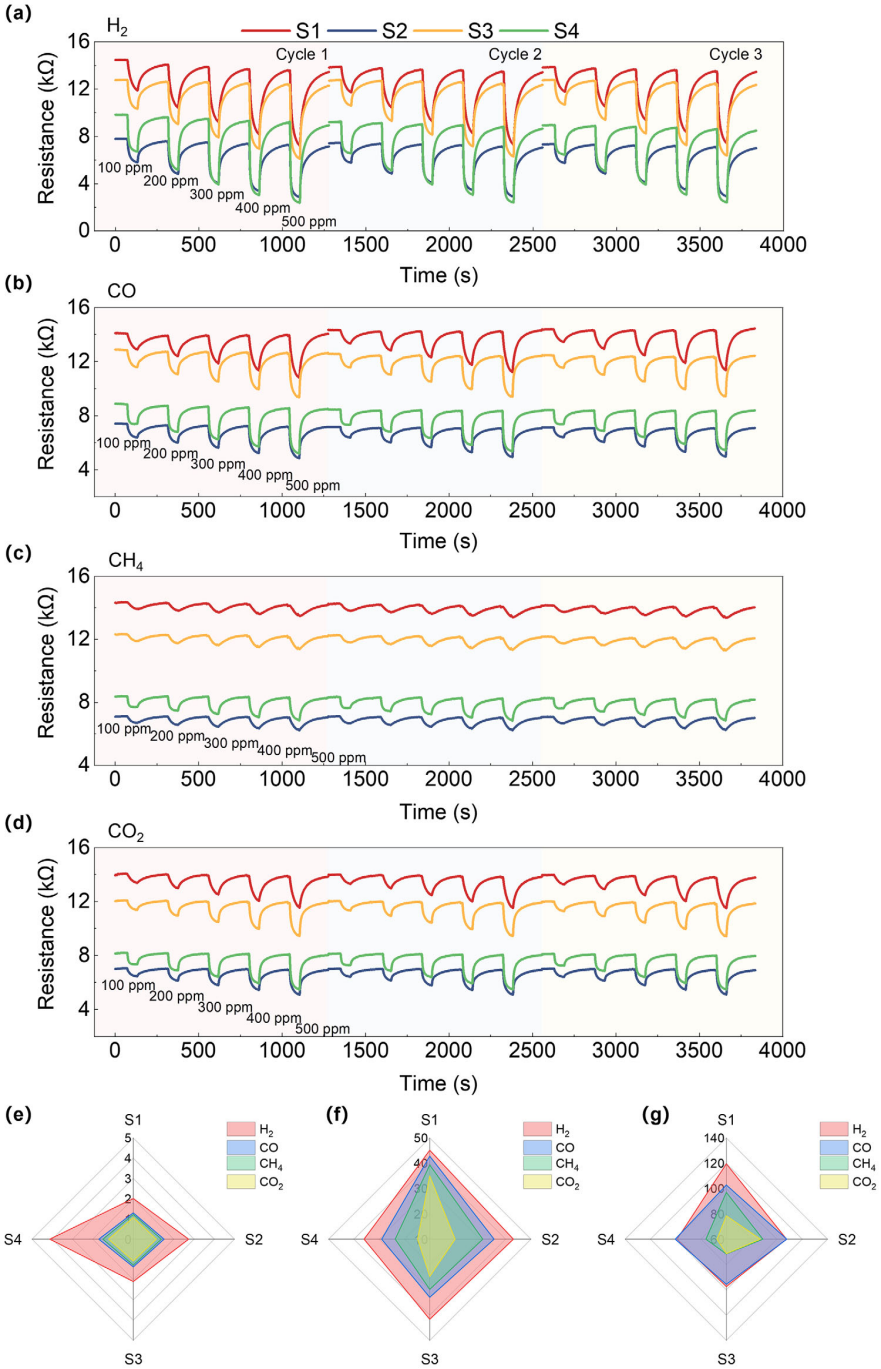
Dynamic response curves of the fresh MEMS sensor array to four types of thermal‐runaway characteristic gases. (a) 100−500 ppm H_2_, (b) 100−500 ppm CO, (c) 100−500 ppm CH_4_, (d) 100−500 ppm CO_2_. Radar plot of 500 ppm CO, CH_4_, CH_4_ and CO_2_: (e) response value, (f) response time, (g) recovery time.

In the gas sensing performance test, the heating voltage for the MEMS sensor array is set at 1.9 V. The corresponding temperatures for each unit are 187°C, 225°C, 244°C, and 259°C, respectively. Figure 2a–d illustrates the dynamic response curves of the fresh single‐chip MEMS gas sensor array, which integrates sensing materials with a microheater array. These data cover three test cycles under exposure to target gases: H_2_, CO, CH_4_, and CO_2_, all of which have been previously reported to be detectable using MOS gas sensors [29, 30, 31, 32]. The response value and response/recovery times of 500 ppm H_2_, CO, CH_4_, and CO_2_ are shown as radar plot in Figure 2e–g, indicating distinct response patterns. Evidently, for a given gas, these parameters vary significantly across the array's different temperature zones. Furthermore, distinct differences are also observed among different target gases on the same sensing unit. These results demonstrate that the single‐chip MEMS gas sensor array is capable of detecting these four critical gases and exhibits distinct temperature‐modulated response patterns.

### 2.2 Feature Construction and Model Design

**FIGURE 3:**
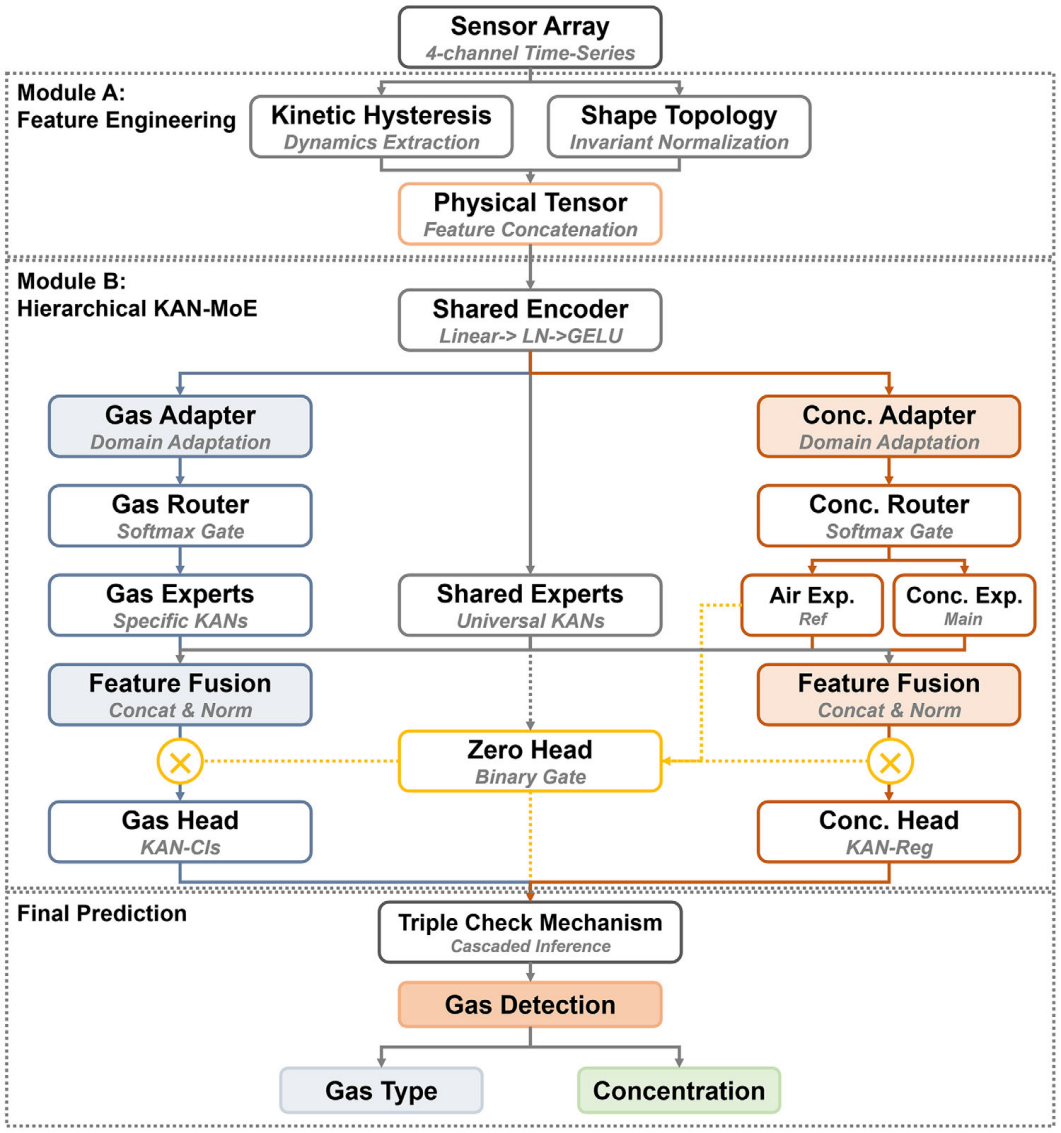
Architecture of the Hierarchical Kolmogorov‐Arnold Mixture‐of‐Experts (HK‐MoE) model.

To address the challenges commonly encountered in gas sensors, including nonlinear response characteristics, sensor drift, and multi‐task learning requirements, this study proposes a learning framework that integrates a physics‐informed dual‐layer feature extraction pipeline with a Hierarchical Kolmogorov‐Arnold Mixture‐of‐Experts (HK‐MoE) model, as illustrated in Figure 3.

#### 2.2.1 Physics‐Informed Feature Engineering

This module establishes a dual‐layer feature extraction pipeline that separates signal intensity (gas concentration) from spatial topology (gas classification).

*Layer 1: Magnitude and Kinetic Descriptors (Drift‐Resilient Intensity)*: The first layer applies a dynamic log‐ratio transformation to the sensor array signals, derived from the Beer‐Lambert law and the Freundlich adsorption model, in order to linearize the power‐law response *S*∝*C^b^* and mitigate baseline drift, as expressed in Equation (1):
(1) $$S\;\left(t\right)=\ln R\left(t\right)\;-\ln R_{\mathrm{base}}\left(t\right)$$

where *R*_base_(*t*) denotes the sliding baseline tracked by an extended high‐quantile filter.

*Layer 2: Topological Shape Descriptors (Invariant Gas Fingerprint)*: The second layer focuses on the shape of the sensor array's response vector, which remains invariant under aging. The log‐ratio response vector is normalized according to Equation (2):
(2) $$\hat{S}\left(t\right)=\frac{S\left(t\right)}{∥S\left(t\right){∥}_{2}}$$

#### 2.2.2 HK‐MoE Model

The HK‐MoE architecture integrates a learnable basis function expansion with a hierarchical gating mechanism to achieve both high expressivity and physical consistency.

##### 2.2.2.1 Kolmogorov‐Arnold Network (KAN) Experts

The model employs KANs in place of conventional Multi‐Layer Perceptrons (MLPs). Each edge in a KAN layer incorporates a learnable spline‐based activation function parameterized by B‐spline basis functions, which is well suited for modeling sensor array responses that inherently follow power‐law transduction and exponential decay kinetics.

##### 2.2.2.2 Hierarchical Routing With Residual Correction

The model employs a residual MoE strategy. A shared encoder first extracts general representations. Task‐specific soft routers then dynamically weight the outputs of specialized KAN experts, where the gating probability encourages each expert to focus on fine‐grained corrections associated with specific gases or concentration ranges without redundantly relearning global features.

##### 2.2.2.3 Zero‐Gate Mechanism

The Zero‐Gate is a specialized head that predicts the probability of “Background Air” (*P*_air_). When activated, the model enforces a zero‐concentration output, thereby suppressing false‐positive predictions caused by baseline drift, as shown in Equation (3):

(3) $${\hat{y}}_{\mathrm{final}}=\left(1-P_{\mathrm{air}}\right){\hat{y}}_{\mathrm{expert}}$$

This mechanism ensures that the system remains silent under no target gas conditions. The gating value is clamped to [0.05, 1.0] to prevent complete suppression of weak gas signals, ensuring robust detection even when the model predicts “Background Air” with high confidence. The full set of training hyperparameters for the HK‐MoE model is provided in Table S3. Table S5 summarizes the main physics‐informed feature blocks used in the HK‐MoE model.

### 2.3 Manifold Drift Analysis

**FIGURE 4:**
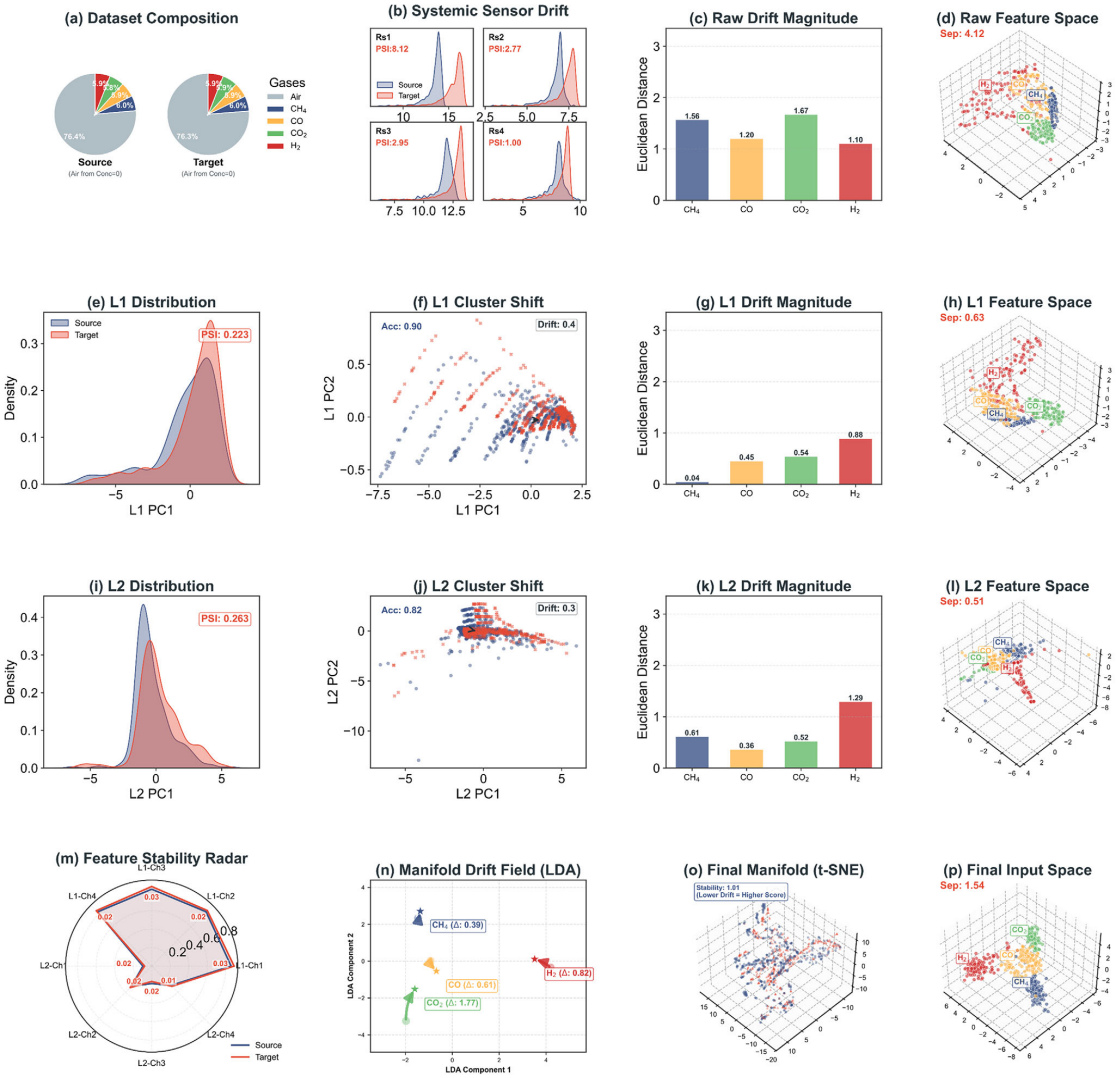
(a) Dataset composition of the Source and Target Domains. (b) The sensor drift across four channels between the two domains in raw feature space. (c) The Euclidean drift distances of the four Target gases across the two domains in raw feature space. (d) The 3D visualization of the raw feature space for the two domains. (e) The sensor drift between the two domains in L1 space. (f) The LDA visualization of cluster shift for the two domains in L1 space. (g) The Euclidean drift distances of the four Target gases across the two domains in L1 space. (h) The 3D visualization of the L1 space for the two domains. (i) The sensor drift between the two domains in L2 space. (j) The LDA visualization of cluster shift for the two domains in L2 space. (k) The Euclidean drift distances of the four Target gases across the two domains in L2 space. (l) The 3D visualization of the L2 space for the two domains. (m) The feature radar plots in the L1 and L2 feature spaces. (n) The manifold drift field of gas clusters across the two domains. (o) The final feature manifold obtained via *t*‐SNE. (p) The 3D visualization of the final input space.

To visualize how physical changes affect the feature space, a comprehensive manifold analysis was conducted on both the Source and Target Domains. Figure 4a presents the composition of the two datasets, where the distribution of gas classes (Background Air, H_2_, CO, CH_4_, and CO_2_) is nearly identical: air accounts for approximately 76%, while each of the four target gases contributes about 6%. The overall drift across the four sensor channels is quantified using the Population Stability Index (PSI), as shown in Figure 4b. All channels exhibit PSI values exceeding 0.5, indicating pronounced drift. The raw Euclidean drift distances of the four target gases across the two domains are shown in Figure 4c. The large drift magnitudes (1.10–1.67) indicate that the raw feature space cannot support reliable gas discrimination. The 3D visualization of the raw feature space in Figure 4d reports an inter‐domain separability index of 4.12 that reflects substantial domain discrepancy and a loss of topological consistency, ultimately limiting model generalization. Figure 4e–l illustrates the drift characteristics in the Log‐Ratio (L1) and Normalized (L2) feature spaces, showing markedly reduced PSI values, drift distances, and separability indexes, respectively. These results demonstrate that the proposed dual‐layer feature extraction pipeline effectively mitigates sensor drift. Figure 4m presents the feature radar plots in the L1 and L2 feature spaces. Although the absolute feature values shift, the relative topological structure among features remains stable. This manifold preservation provides a reliable physical foundation for the HK‐MoE model, enabling it to leverage stable experts while down‐weighting drift‐prone channels. Figure 4n shows the manifold drift field of gas clusters across the two domains, where LDA maps the cluster centers from the Source to the Target Domain. The drift magnitude (Δ) exhibits clear gas dependence, with CH_4_ being the most stable (Δ = 0.39) and CO_2_ exhibiting the largest displacement (Δ = 1.77). The final feature manifold obtained via *t*‐SNE is shown in Figure 4o, revealing well‐preserved topological separation. The 3D visualization of the final input space in Figure 4p further confirms successful alignment between the Source and Target Domains, ensuring robust inputs for the HK‐MoE model.

### 2.4 Multi‐Gas Discrimination With Deep Learning Algorithm

**FIGURE 5:**
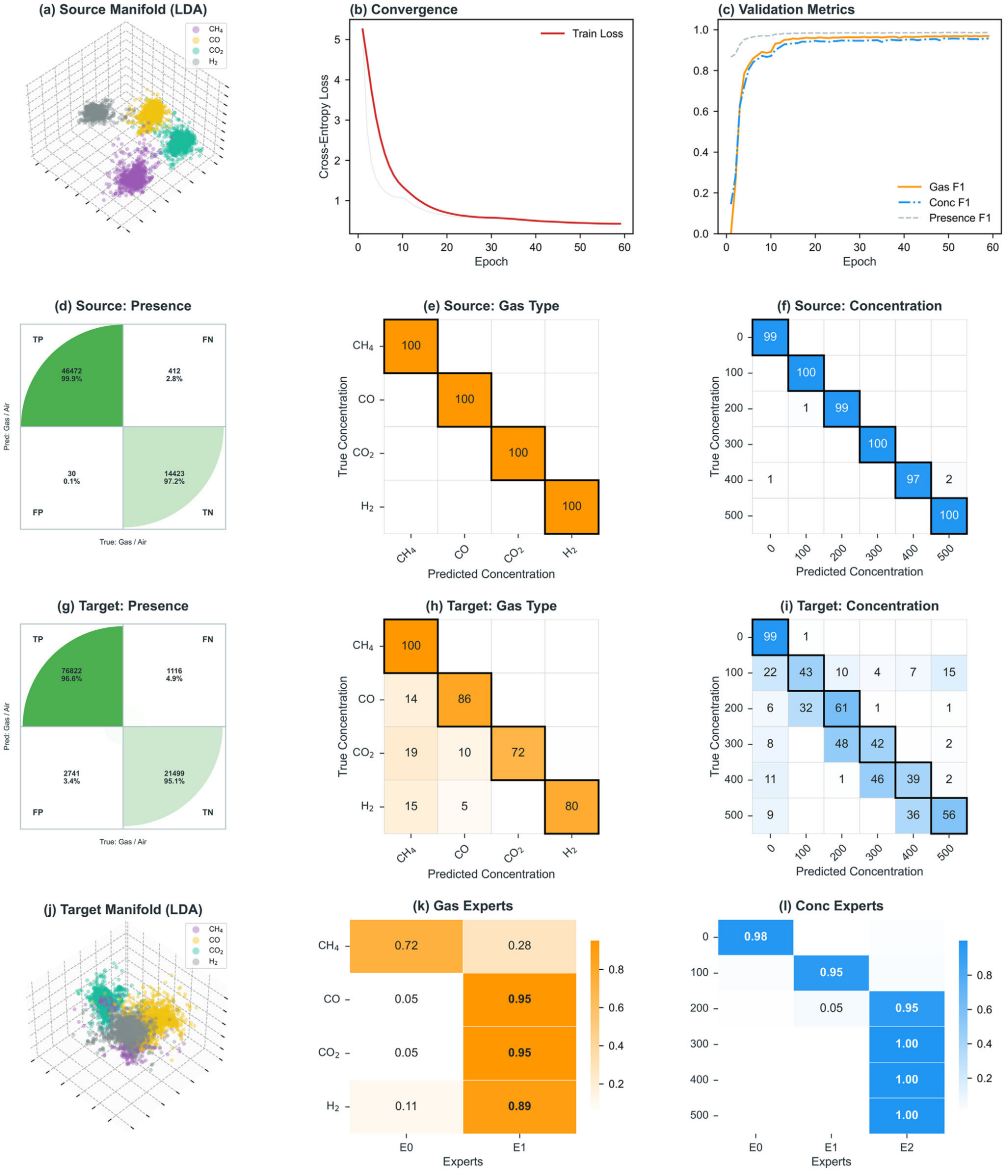
(a) The LDA visualization of the learned features on Source Domain. (b) The cross‐entropy loss curve. (c) The multi‐task validation curves. The confusion matrices for (d) target gas presence detection, (e) gas classification, and (f) concentration determination on Source Domain. The confusion matrices for (g) target gas presence detection, (h) gas classification, and (i) concentration determination on Target Domain. (j) The LDA visualization of learned features on Target Domain. The activation heatmaps of the (k) gas experts and (l) concentration experts.

On the Source Domain, the HK‐MoE model demonstrates excellent performance. Figure 5a presents the LDA visualization of the learned features, where the clusters of the four target gases are clearly separated with minimal overlap. This indicates that the combined framework successfully captures highly discriminative gas characteristics. The cross‐entropy loss curve in Figure 5b decreases monotonically and converges within approximately 40 epochs without noticeable oscillation, suggesting a stable optimization process with no signs of overfitting or divergence. The multi‐task validation curves in Figure 5c indicate that all three tasks achieve near‐perfect performance, including target gas presence detection (Pres. F1 = 99.53%), gas classification (Class. F1 = 99.88%), and concentration determination (Conc. F1 = 98.09%), as summarized in Table 1. The confusion matrices in Figure 5d–f further confirm the high accuracy and reliability of the model across all tasks.

**TABLE 1:** Task‐wise F1‐scores on the Source and Target Domains.

Dataset	Pres. F1	Class. F1	Conc. F1
Source Domain	99.53%	99.88%	98.09%
Target Domain	97.55%	80.62%	57.68%

On the Target Domain, the HK‐MoE model maintains a solid degree of robustness against sensor drift and continues to deliver satisfactory performance. As shown in Table 1, target gas presence detection remains highly reliable (Pres. F1 = 97.55%), while gas classification (Class. F1 = 80.62%) and concentration determination (Conc. F1 = 57.68%) exhibit moderate degradation. The confusion matrices in Figure 5g–i reveal that target gas presence detection remains accurate. For gas classification, CH_4_ is still identified reliably, while CO, CO_2_, and H_2_ exhibit noticeable cross‐class confusion. For concentration determination, predictions remain relatively accurate at low concentrations (100 ppm), whereas more pronounced confusion appears at higher concentration levels. Although overall performance decreases on the Target Domain, the decision boundaries remain stable and the model significantly outperforms traditional baseline models (LSTM: Class. F1 = 35.50%, Conc. F1 = 43.54%; LightGBM: Class. F1 = 25.63%, Conc. F1 = 51.92%) and state‐of‐the‐art Transformer‐based time‐series models (iTransformer: Class. F1 = 30.19%, Conc. F1 = 44.57%; PatchTST: Class. F1 = 19.09%, Conc. F1 = 35.02%), as summarized in Table S6. The experimental setup and hyperparameter configurations for iTransformer and PatchTST models are provided in Table S4.

The performance degradation could arise from several established aging mechanisms of MOS sensing materials under prolonged high‐temperature operation, including grain growth and variations in oxygen vacancy concentration [33, 34]. In addition, long‐term operation of the MEMS microheater may induce changes in heater resistance, thermal stress accumulation, and slight variations in the temperature distribution across the sensing units [35]. These factors may collectively contribute to the observed drift in sensor resistance and response, which in turn can degrade model performance over prolonged operation.

Figure 5j displays the LDA distribution of learned features on Target Domain, further indicating that the model successfully preserves domain‐invariant structure, allowing gas clusters to remain separable despite covariate shift. Figure 5k,l shows the activation heatmaps of the gas experts and concentration experts, revealing the internal decision mechanisms of the model. Different KAN experts are selectively activated for specific gas types or concentration ranges, demonstrating that the HK‐MoE architecture effectively decomposes the complex sensing task into manageable subproblems and achieves a coherent integration of task specialization and physical consistency.

### 2.5 Current Limitations, Technology Readiness, and Future Perspectives

#### 2.5.1 Ambient Humidity and Temperature Effects

**FIGURE 6:**
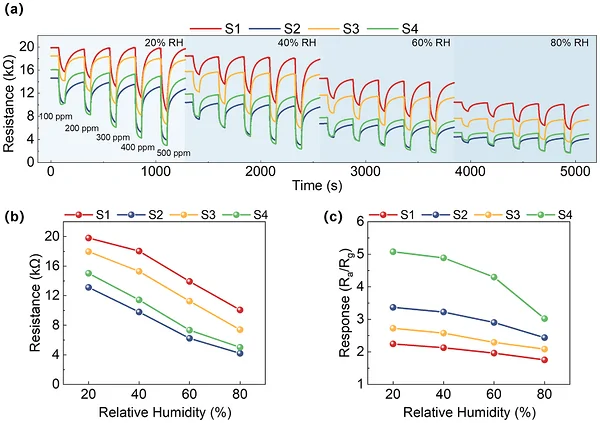
(a) Dynamic response curves of the MEMS sensor array to 100−500 ppm H_2_ under different ambient humidity conditions (20, 40%, 60%, and 80% RH) at an ambient temperature of 25°C. (b) Baseline resistance of the MEMS sensor array to 500 ppm H_2_ under different ambient humidity conditions (20, 40%, 60%, and 80% RH) at an ambient temperature of 25°C. (c) Response of the MEMS sensor array to 500 ppm H_2_ under different ambient humidity conditions (20, 40%, 60%, and 80% RH) at an ambient temperature of 25°C.

To investigate the humidity‐dependent gas sensing performance of the MEMS sensor array, the dynamic response curves of the array when exposed to 100–500 ppm H_2_ under different ambient relative humidity (RH) conditions (20, 40%, 60%, and 80%) at an ambient temperature of 25°C were measured, as shown in Figure 6a. Figure 6b,c displays the corresponding baseline resistance and response values of the array to 500 ppm H_2_ under different humidity conditions, respectively. Both the baseline resistance and the response values decreased monotonically with increasing RH. As the RH increased from 20% to 80%, the baseline resistance and response values across the four sensing units decreased by 49.14%–67.99% and 21.88%–40.48%, respectively. This phenomenon can be attributed to competitive adsorption of water molecules on the surface of the SnO_2_ nanoparticles, which reduces the available active sites for oxygen and target gas adsorption. Consequently, the baseline resistance is lowered, accompanied by a reduction in the sensor response [36].

The influence of ambient temperature on the sensing performance of the array to 500 ppm H_2_ is illustrated in Figure S8. As the ambient temperature rose from 25°C to 60°C, the baseline resistance and response values across the four sensing units decreased slightly by 28.95%–46.63% and 4.42%–22.49%, respectively. This behavior can be explained by the reduced temperature gradient between the microheater and the surrounding air as ambient temperature increases, which suppresses heat dissipation. Consequently, under a constant heating voltage, the actual operating temperature of the microheater increases slightly, thereby causing the observed minor reductions in baseline resistance and response. The effects of ambient humidity and temperature on the sensing performance to CO, CH_4_, and CO_2_ are presented in Figures S9–S11. Similar to the trend observed for H_2_, both the baseline resistance and response values of all four sensing units decreased with increasing ambient RH and temperature.

We recognize that variations in ambient temperature and humidity can affect the sensing performance of the proposed system. In future work, environmental temperature and humidity compensation strategies will be incorporated to reduce these influences and enhance the system's robustness under practical operating conditions.

#### 2.5.2 Device‐to‐Device Variation

To demonstrate the reproducibility and consistency across multiple devices, five additional devices were fabricated and tested under identical experimental conditions (making a total of six MEMS sensor array devices, denoted as #1 to #6). Figure S12a,b displays the dynamic response curves of the six devices to 100−500 ppm H_2_, while the corresponding baseline resistance and response values to 500 ppm H_2_ are presented in Figure S12c,d. Notably, the relative response patterns among the four sensing units (S1−S4) remained highly consistent across all six devices. Specifically, the S1 unit consistently exhibited the highest baseline resistance, while the S4 unit consistently showed the highest response value. This indicates that the temperature‐dependent sensing characteristics of the array were well preserved among independently fabricated devices.

To quantitatively evaluate the device‐to‐device fluctuations, the coefficient of variation (CV) for the baseline resistance and response values of each unit was calculated across the six devices, as summarized in Tables S1 and S2. The CVs of the baseline resistance ranged from 7.01% to 10.53%, while those of the response values ranged from 5.38% to 18.93%. These relatively low CV values are all well within the acceptable range of typical microfabrication and materials processing variations, demonstrating good fabrication reproducibility and device‐to‐device consistency of the proposed MEMS sensor array.

#### 2.5.3 Long‐Term Sensor Drift

To further evaluate the long‐term stability and drift performance of the proposed system, the device previously aged for 30 days was subjected to an additional 30 days of continuous aging under the same operating conditions. Figure S7 presents the dynamic response curves of the MEMS sensor array after a total of 60 days of aging under exposure to 100–500 ppm of H_2_, CO, CH_4_, and CO_2_. Compared to the fresh device and the 30‐day aged device results shown in Figures 2a–d and S6, the baseline resistances of the 60‐day aged sensor array exhibit a distinct increasing trend, accompanied by a decrease in their response values.

Under the 60‐day aging condition, the HK‐MoE model achieves F1‐scores of 94.83%, 53.59%, and 30.65% for target gas presence detection, gas classification, and concentration determination, respectively, indicating that prolonged sensor drift primarily degrades gas classification and concentration determination. Notably, the model still maintains reliable target gas presence detection, which is the primary requirement for LIBs thermal runaway early warning.

To mitigate the performance degradation caused by long‐term sensor drift, we further investigated a few‐shot calibration strategy (Figures S13 and S14). As summarized in Table S7, by employing the concentration‐balanced sampling strategy with only 100 labeled samples (*k* = 100), the F1‐scores for gas classification and concentration determination on the 60‐day aging dataset recovered to 83.50% and 72.92%, respectively. These results demonstrate that the proposed calibration method effectively restores the degraded model performance using only a small number of labeled calibration samples, highlighting the potential of combining the HK‐MoE framework with few‐shot calibration for long‐term field deployment.

However, we agree that further validation over substantially longer aging periods is still required before practical deployment. Therefore, the present work should be regarded as an initial investigation of long‐term sensor drift rather than a comprehensive lifetime validation. Future work will include aging experiments over several months or even years, combined with periodic recalibration, online drift compensation, and sensing‐material optimization to further improve the long‐term robustness and reliability of the proposed framework.

#### 2.5.4 Mixed‐Gas Operation

**FIGURE 7:**
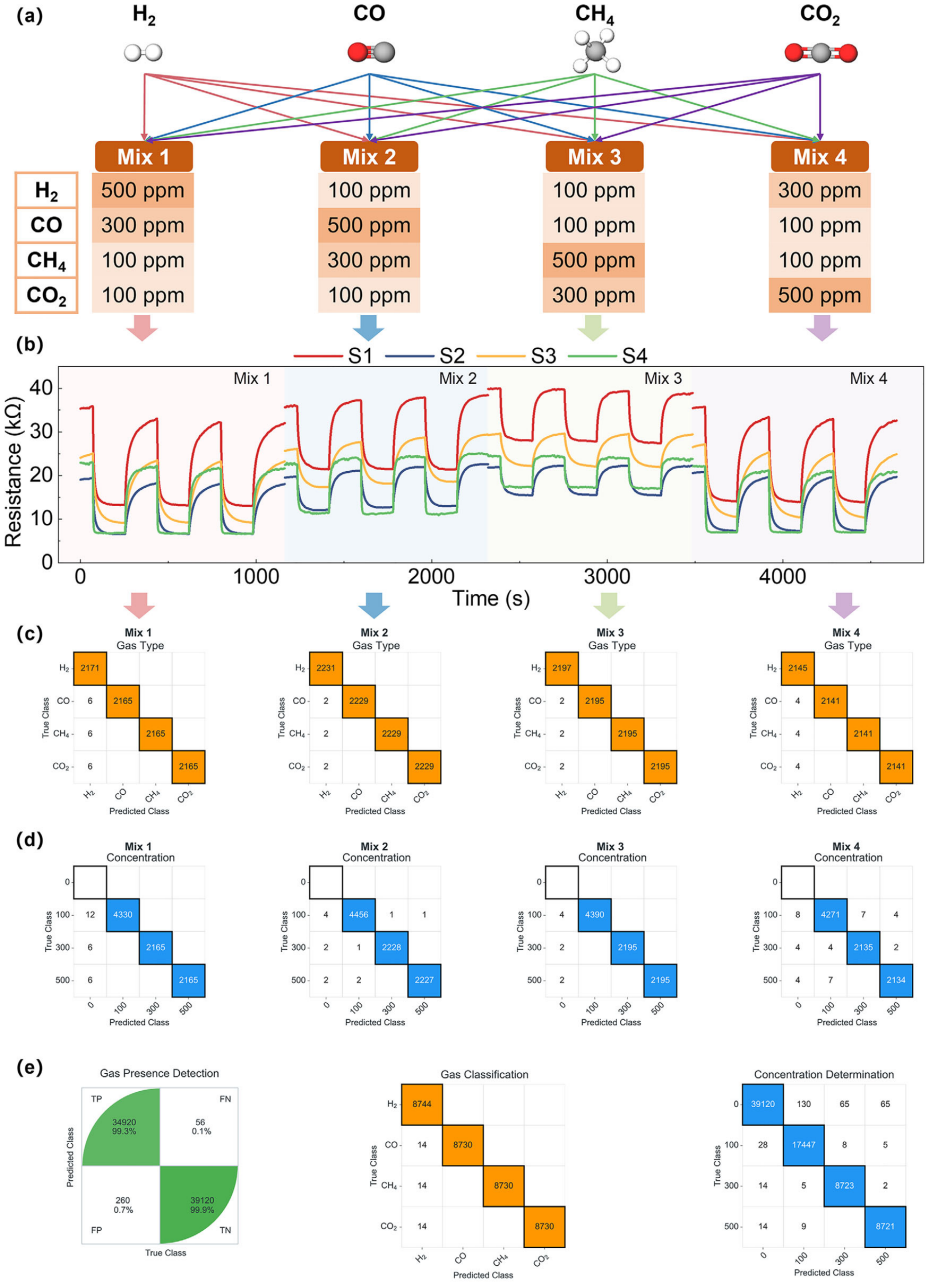
(a) Experimental design of gas mixture samples. (b) Response curves of the MEMS sensor array to four multi‐component gas samples (Mix 1, Mix 2, Mix 3, Mix 4). The confusion matrices for (c) gas classification and (d) concentration determination of the individual multi‐component gas samples. (e) Overall confusion matrices for target gas presence detection, gas classification, and concentration determination using the complete multi‐component gas dataset.

To further prove the application potential of the proposed system, mixed‐gas experiments were performed to showcase its capability for deconvolving mixed‐gas signatures. Specifically, four characteristic gases (H_2_, CO, CH_4_, and CO_2_) were blended at different concentrations into four multi‐component gas samples (Mix 1, Mix 2, Mix 3, Mix 4), with the total concentration maintained at 1000 ppm. Figure 7a illustrates the experimental design of gas mixture samples. The dynamic response curves of the single‐chip MEMS sensor array toward the four multi‐component gas samples are illustrated in Figure 7b. Notably, the baseline resistances of all sensing units increased compared to those observed in Figure 2. This systematic upward shift can be attributed to the altered gas distribution systems. Specifically, while a flow rate of 100 sccm was used for single‐gas evaluations, a larger recommended flow rate of 1000 sccm was adopted for the mixed‐gas experiments to ensure rapid and uniform mixing of the component gases.

Figure 7c,d presents the confusion matrices for gas classification and concentration determination of the individual multi‐component gas samples, respectively. For all four gas mixtures, the proposed HK‐MoE model achieved consistently high recognition accuracy with only a few misclassifications. As summarized in Table S8, the F1‐scores for target gas presence detection, gas classification, and concentration determination all exceeded 99.62%, demonstrating excellent discrimination capability under different mixed‐gas compositions. Figure 7e displays the overall confusion matrices for the three tasks across the complete multi‐component gas dataset. The HK‐MoE model achieved overall Pres. F1, Class. F1, and Conc. F1‐scores of 99.92%, 99.88%, and 99.83%, respectively (Table S8), confirming its robust performance across all mixed‐gas samples.

As summarized in Table S9, the Class. F1‐scores for H_2_, CO, CH_4_, and CO_2_ all exceeded 99.72%, while the corresponding Conc. F1‐scores reached 99.86%, 99.86%, 99.86%, and 99.83%, respectively. These results demonstrate that the proposed framework can effectively decouple the overlapping responses of multiple characteristic gases and accurately identify both the dominant gas species and its concentration in complex multi‐component gas mixtures.

To better illustrate the advancements of the proposed framework, a comprehensive comparison with state‐of‐the‐art gas sensing systems for LIBs thermal runaway monitoring is presented in Table S10. The comparison demonstrates that the proposed framework offers several distinctive advantages over existing studies, including a self‐made miniaturized single‐chip integrated MEMS sensor array, competitive multi‐component gas classification and concentration estimation performance, and a dedicated long‐term aging evaluation of sensor drift alongside a few‐shot calibration strategy to effectively restore performance, all of which contribute to its potential for practical deployment.

Overall, although the proposed framework demonstrates promising sensing performance under controlled laboratory conditions, further investigation is required before practical deployment, particularly regarding environmental robustness, long‐term stability, and validation under realistic LIB thermal runaway scenarios.

## 3 Conclusion

This work presents a 2 × 2 integrated MEMS gas sensor array with spatial temperature modulation and a deep‐learning framework for identifying thermal‐runaway characteristic gases. The SnO_2_‐functionalized array produces distinct temperature‐modulated response patterns for H_2_, CO, CH_4_, and CO_2_, enabling reliable multi‐gas analysis when combined with the proposed HK‐MoE model. Under single‐gas conditions, the proposed model deployed on a fresh sensor array achieved F1‐scores of 99.53% for target gas presence detection, 99.88% for gas classification, and 98.09% for concentration determination. A30‐day continuous operational aging study further demonstrated satisfactory drift resilience, with the model maintaining F1‐scores of 97.55%, 80.62%, and 57.68% for the three tasks, respectively, outperforming conventional baselines and state‐of‐the‐art Transformer‐based time‐series models. Although model performance noticeably degraded after 60 days of aging, the proposed few‐shot calibration strategy effectively restored F1‐scores with small number of labeled calibration samples (*k* = 100), confirming its practical feasibility for long‐term deployment. Furthermore, the system achieved overall F1‐scores exceeding 99.83% across all three tasks on the complete multi‐component dataset, highlighting its exceptional capability in mixed‐gas discrimination. Overall, these results highlight the promise of combining spatial temperature modulation, physics‐informed feature engineering, and the HK‐MoE architecture to build robust early‐warning gas sensing systems for battery safety, with future work extending toward real‐world field validation.

## 4 Experimental Section

### 4.1 Chemicals and Raw Materials

All chemicals and reagents were purchased from Sigma–Aldrich and can be used without further purification, including SnCl_4_·5H_2_O, 37 wt.% HCl, 30 wt.% H_2_O_2_, 25 wt.% aqueous ammonia, 50 wt.% KOH etchant, ethylene glycol. The n‐type silicon wafer with a crystal face of (100) and a thickness of 500 µm was purchased from Suzhou Research Materials Microtech Co., Ltd. All gases used for gas sensing performance test, including dry air, 1000 ppm H_2_, 1000 ppm CO, 1000 ppm CH_4_, and 1000 ppm CO_2_, were purchased from Shenzhen Huashidai Technology Co., Ltd. Each target gas sample consists of the dry target gas and nitrogen, prepared to achieve the specified concentration provided by the supplier.

### 4.2 Fabrication of Single‐Chip MEMS Gas Sensor Array

#### 4.2.1 Synthesis of Gas Sensing Materials

The SnO_2_ sensing material was synthesized via a solution‐based method. Briefly, 1.5 g of SnCl_4_·5H_2_O was dissolved in 60 mL of deionized (DI) water under ultrasonication. The solution was heated to 65°C in an oil bath and stirred for 1 h, followed by an additional 15 min of stirring outside the bath to ensure homogeneity. The precipitate was separated by centrifugation at 3000 rpm for 3 min and dried overnight at 100°C. To prepare the sensing ink, 0.8 g of the dried powder was ball‐milled with 20 mL of ethylene glycol and 4 g of zirconia beads using a planetary ball mill (Pulverisette 7, FRITSCH, Germany).

#### 4.2.2 Device Fabrication and Packaging

The 2 × 2 single‐chip MEMS gas sensor array comprises a substrate, a supporting layer, heating electrodes, an insulating layer, detecting electrodes, and the gas sensing material, as shown in Figure S1. A novel fence‐shaped electrode structure integrates the connections for all heating and sensing electrodes, reducing the pin count and enabling the simultaneous formation of four distinct heating regions. The detailed layout design and fabrication process flow are presented in Figures S2 and S3, respectively. The sensor array was functionalized by dispensing the sensing ink onto the microheater surface, followed by annealing in air at 600°C for 5 min and subsequently at 450°C for 4 h. The fabricated device was wire‐bonded and packaged in a ceramic shell (5 × 5 × 1 mm^3^). Finally, a metal lid with seven gas vents was attached to protect the sensor array while ensuring gas accessibility.

### 4.3 Measurement and Characterization

The image of the MEMS microheater was observed using an optical microscope (BX51, OLYMPUS, Japan). The thermal performance of the MEMS microheater was measured using an infrared thermal imaging instrument (Image IR 8300, INFRATEC, Germany). The resistances of the heating electrodes were tested by probe station (Summit 11000B‐M, Cascade, USA) and source meter (4200, KEITHLEY, USA). The crystallinity of gas sensing materials was estimated using x‐ray diffraction (XRD, SmartLab, Rigaku, Japan). The morphological characteristics of gas sensing materials were studied using scanning electron microscopy (SEM, Merlin, ZEISS, Germany). The elemental compositions and surface chemical states of gas sensing materials were determined using x‐ray photoelectron spectroscopy (XPS, Xi+, Escalab, USA). Figure S4 presents a schematic of the test circuit used to measure the resistance of the gas sensor array.

Single‐gas sensing tests were conducted using a gas sensing test system (JF02F, Guiyan Jinfeng Co., Ltd., China) with a flow rate of 100 sccm, as shown in Figure S5. Mixed‐gas sensing tests were conducted using a gas and liquid distribution system (DGD‐VI, SINOAGG, China) with a flow rate of 1000 sccm to ensure rapid and uniform mixing of the component gases. Unless otherwise noted, all gas sensing measurements were performed at an ambient temperature of 25°C and an RH of 60%. The ambient temperature‐dependent and humidity‐dependent gas sensing performance were evaluated using a constant temperature and humidity test chamber (WHTH‐408L‐0‐880, WEWON, China). The sensor response is defined as the ratio of R_a_ (resistance in air) to R_g_ (resistance in the target gas).

### 4.4 Dataset Collection

To comprehensively evaluate the model's robustness against sensor drift, two datasets were collected from the single‐chip MEMS MOS sensor array under distinct aging conditions. The Source Domain (*D_S_*) dataset contains 306 679 samples acquired from the fresh array across three measurement cycles for each target gas (H_2_, CO, CH_4_, and CO_2_) within a concentration range of 100–500 ppm, as shown in Figure 2a–d. *D_S_* was utilized for model training and initial performance evaluation. The Target Domain (*D_T_*) dataset contains 102,178 samples collected from the same array after 30 days of aging across one measurement cycle at identical concentration levels, as shown in Figure S6. *D_T_* captured the drift characteristics of the aged array and was used exclusively for model evaluation and few‐shot calibration to assess generalization. In both datasets, gas concentrations follow a discrete step pattern (100, 200, 300, 400, 500 ppm). The “Background Air” class, defined as the baseline condition (0 ppm target gas), was inserted between gas injection cycles to capture recovery dynamics. Data were sampled at 20 Hz, corresponding to a 50 ms interval.

## Author Contributions

**Qin Luo**: data curation, writing – original draft, visualization, investigation, validation. **Qi Guo**: writing – original draft, visualization, formal analysis, investigation, validation. **Rongyue Liu**: conceptualization, methodology, writing – review and editing. **Yilin Li**: investigation. **Haiping Zhu**: investigation. **Wei Zhang**: investigation. **Zhongren Chen**: resources. **Zhaojun Liu**: resources. **Xing Cheng**: supervision, funding acquisition, project administration, writing – review and editing, conceptualization.

## Funding

This work is financially supported in part by Shenzhen Science and Technology Innovation Committee (Grant No. RCJC20200714114436046), National Natural Science Foundation of China (Grant No. 31927802), Key research and development projects in Xizang Autonomous Region (Grant Nos. XZ202601ZY0174, SUSTECHJX2022007).

## Conflicts of Interest

The authors declare no conflicts of interest.

## Supporting information




**Supporting File**: advs77784‐sup‐0001‐SuppMat.docx.

## Data Availability

The data that support the findings of this study are available from the corresponding author upon reasonable request.
