# Supplementary material for: Temperature‐Modulated MEMS Gas Sensor Array Enabled by Physics‐Informed Deep Learning for Thermal‐Runaway Characteristic Gas Discrimination
Source: Adv Sci (Weinh). 2026 Sep 16:e77784. Online ahead of print. doi: 10.1002/advs.77784 (PMC13583223; doi:10.1002/advs.77784)
Supplement: Supplementary file 1 [file ADVS-9999-e77784-s001.docx]

Supporting Information

Temperature‑Modulated MEMS Gas Sensor Array Enabled by Physics‑Informed Deep Learning for Thermal‑Runaway Characteristic Gas Discrimination

*Qin Luo, Qi Guo, Rongyue Liu*, Yilin Li, Haiping Zhu, Wei Zhang, Zhongren Chen, Zhaojun Liu, Xing Cheng**

**1. Design and Fabrication of Single-Chip MEMS Gas Sensor Array**

**Figure S1.** Schematic of the structure of the MEMS gas sensor array.

**Figure S2.** The layout design of (a) the MEMS microheater array (three aligned layout), (b) heating electrodes, (c) detecting electrodes, (d) etching windows.

**Figure S3.** The fabrication process of the MEMS microheater.

The silicon wafer was first cleaned sequentially in HCl/H_2_O_2_, NH_3_/H_2_O_2_, and deionized (DI) water, followed by drying with nitrogen gas. A 1 μm low-stress silicon nitride (SiN_x_) supporting layer was then deposited via low-pressure chemical vapor deposition (LPCVD). Subsequently, the wafer was patterned, and a 215-nm-thick Ti/Pt (15/200 nm) layer was deposited by sputtering to form the heating electrodes. A 450-nm-thick SiN_x_ layer was subsequently deposited as an insulating layer via plasma-enhanced chemical vapor deposition (PECVD). This insulating layer was etched using inductively coupled plasma (ICP) to expose the heating electrode pads. The wafer was then patterned again, and a second 215-nm-thick Ti/Pt (15/200 nm) layer was sputtered to form the detecting electrodes. Next, the supporting SiNₓ layer was etched via ICP to create windows for wet etching. Finally, the wafer was etched in 40 wt% KOH at 70 °C for 3 h to release the suspended structure and subsequently cleaned with DI water.

**2. Measurement of Single-Chip MEMS Gas Sensor Array**

**2.1 Structure of Test Circuit**

**Figure S4.** Schematic of the test circuit used to measure the resistance of the gas sensor array.

In this circuit, V_D_ represents the detecting voltage, R_1_ to R_4_ are four matching resistors, and R_S1_ to R_S4_ denote the resistance of the gas sensing material on each sensor.

**2.2 Working Principle of Gas Sensing Test System**

**Figure S5.** Schematic of the gas sensing test system.

The working principle of gas sensing test system (JF02F) is described as follows. After passing through the mass flow controller (MFC), one type of target gas and dry air enter the gas distributing chamber, where dry air is divided into two streams (dry air1 and dry air2). Dry air1 acts as the background gas, while dry air2 is employed to blend with target gas, thereby reducing its concentration. The flow rates for the three streams and the switching of gas line are modulated by computer. During the test phase, the mixture of target gas and dry air2 is directed to the test chamber, while dry air1 is routed there before or after the test. The concentration of the diluted target gas (*C_target_*) is calculated as **Equation S1**:

$C_{target}=\frac{F_{target}}{F_{da1}}\cdot C_{initial}$	(S1)

where *F_target_* is the flow rate of target gas, *F_da1_* is the flow rate of dry air1, *C_initial_* is the initial target gas concentration provided by the supplier. By maintaining the combined flow rates of the target gas and dry air2 equal to that of dry air1, fluctuations in sensor resistance due to gas line switching can be effectively minimized.

**2.3 Aging Protocol**

The sensor array devices were subjected to a continuous operational aging protocol under controlled laboratory conditions, maintained at an ambient temperature of 25 °C and an indoor RH of 60%. Throughout the aging period, a constant 100 sccm flow of dry air was supplied while a continuous heating voltage of 1.9 V was applied to the microheater array.

**3. Gas Sening Experimental Results**

**Figure S6.** The Target Domain dataset (after 30 days of continuous operational aging).

**Figure S7.** Dynamic response curves of the MEMS sensor array after a total of 60 days of aging under exposure to 100–500 ppm of H_2_, CO, CH_4_, and CO_2_.

**Figure S8.** (a) Dynamic response curves of the MEMS sensor array to 100−500 ppm H_2_ under different ambient temperature conditions (25, 40, 50, and 60 °C) at 60% RH. (b) Baseline resistance of the the MEMS sensor array to 500 ppm H_2_ under different ambient temperature conditions (25, 40, 50, and 60 °C) at 60% RH. (c) Response of the the MEMS sensor array to 500 ppm H_2_ under different ambient temperature conditions (25, 40, 50, and 60 °C) at 60% RH.

**Figure S9.** (a) Dynamic response curves of the MEMS sensor array to 100−500 ppm CO under different ambient humidity conditions (20, 40, 60, and 80% RH) at an ambient temperature of 25 °C. (b) Baseline resistance of the MEMS sensor array to 500 ppm CO under different ambient humidity conditions. (c) Response of the the MEMS sensor array to 500 ppm CO under different ambient humidity conditions. (d) Dynamic response curves of the MEMS sensor array to 100−500 ppm CO under different ambient temperature conditions (25, 40, 50, and 60 °C) at 60% RH. (e) Baseline resistance of the the MEMS sensor array to 500 ppm CO under different ambient temperature conditions. (f) Response of the the MEMS sensor array to 500 ppm CO under different ambient temperature conditions.

**Figure S10.** (a) Dynamic response curves of the MEMS sensor array to 100−500 ppm CH_4_ under different ambient humidity conditions (20, 40, 60, and 80% RH) at an ambient temperature of 25 °C. (b) Baseline resistance of the MEMS sensor array to 500 ppm CH_4_ under different ambient humidity conditions. (c) Response of the the MEMS sensor array to 500 ppm CH_4_ under different ambient humidity conditions. (d) Dynamic response curves of the MEMS sensor array to 100−500 ppm CH_4_ under different ambient temperature conditions (25, 40, 50, and 60 °C) at 60% RH. (e) Baseline resistance of the the MEMS sensor array to 500 ppm CH_4_ under different ambient temperature conditions. (f) Response of the the MEMS sensor array to 500 ppm CH_4_ under different ambient temperature conditions.

**Figure S11.** (a) Dynamic response curves of the MEMS sensor array to 100−500 ppm CO_2_ under different ambient humidity conditions (20, 40, 60, and 80% RH) at an ambient temperature of 25 °C. (b) Baseline resistance of the MEMS sensor array to 500 ppm CO_2_ under different ambient humidity conditions. (c) Response of the the MEMS sensor array to 500 ppm CO_2_ under different ambient humidity conditions. (d) Dynamic response curves of the MEMS sensor array to 100−500 ppm CO_2_ under different ambient temperature conditions (25, 40, 50, and 60 °C) at 60% RH. (e) Baseline resistance of the the MEMS sensor array to 500 ppm CO_2_ under different ambient temperature conditions. (f) Response of the the MEMS sensor array to 500 ppm CO_2_ under different ambient temperature conditions.

**Figure S12.** (a), (b) Dynamic response curves of six MEMS sensor array devices to 100−500 ppm H_2_. (c) Baseline resistance of the six MEMS sensor array devices to 500 ppm H_2_. (d) Response values of the six MEMS sensor array devices to 500 ppm H_2_.

**Table S1.** The baseline resistance of each unit across the six devices to 500 ppm H_2_.

| Device | S1 (kΩ) | S2 (kΩ) | S3 (kΩ) | S4 (kΩ) |
| --- | --- | --- | --- | --- |
| #1 | 13.541 | 7.111 | 12.505 | 8.611 |
| #2 | 15.457 | 6.007 | 12.071 | 6.489 |
| #3 | 13.999 | 6.332 | 10.825 | 7.048 |
| #4 | 15.786 | 5.785 | 9.18 | 7.221 |
| #5 | 12.936 | 5.947 | 9.907 | 8.138 |
| #6 | 14.441 | 6.784 | 11.186 | 8.185 |
| Mean | 14.360 | 6.328 | 10.946 | 7.615 |
| Standard Deviation  (SD) | 1.006 | 0.477 | 1.152 | 0.746 |
| Coefficient of Variation  (CV) | 7.01% | 7.54% | 10.53% | 9.79% |

*$\text{CV}\text{=}\frac{\text{SD}}{\text{Mean}}\text{×100\%}$

**Table S2.** The response values of each unit across the six devices to 500 ppm H_2_.

| Device | S1 | S2 | S3 | S4 |
| --- | --- | --- | --- | --- |
| #1 | 1.831 | 2.434 | 1.962 | 3.566 |
| #2 | 1.757 | 1.854 | 1.789 | 2.197 |
| #3 | 1.847 | 2.741 | 2.098 | 4.274 |
| #4 | 1.664 | 1.923 | 1.782 | 3.348 |
| #5 | 1.687 | 2.366 | 2.072 | 3.193 |
| #6 | 1.585 | 2.355 | 2.043 | 3.062 |
| Mean | 1.729 | 2.279 | 1.958 | 3.273 |
| Standard Deviation  (SD) | 0.093 | 0.305 | 0.129 | 0.620 |
| Coefficient of Variation  (CV) | 5.38% | 13.39% | 6.58% | 18.93% |

**4. Data Processing Pipeline**

All machine-learning experiments are conducted using datasets generated by the physics-informed preprocessing pipeline implemented in the project scripts (e.g., Data_Engineering/data_processor.py and Data_Engineering/feature_extractor.py). Raw Source Domain and Target Domain sequences are first cleaned, time-ordered, and if enabled, processed by a physics-based label-correction module that simulates exponential decay during chamber flushing to avoid mislabeling residual gas as Target Gas Presence. The cleaned sequences are then passed to FeatureBuilderV3, which constructs a hybrid feature representation combining log-ratio magnitude features referenced to an adaptive high-quantile baseline, shape-normalized vectors, statistical descriptors and kinetic/hysteresis descriptors capturing adsorption and recovery dynamics.

For the main experiments, the Source Domain dataset is split into training and test subsets using either stratified random sampling or a stratified time-block strategy that preserves temporal ordering within each gas–concentration combination. A statistics layer is fitted on the training subset and reused for all other splits to avoid information leakage.

The resulting feature matrices and labels are exported as standardized CSV files (train_set.csv, test_set.csv, target_domain_set.csv), accompanied by a feature definition file and a log of the split configuration, enabling full reproducibility of the HK-MoE training and evaluation protocol.

**Table S3.** Training hyperparameters for HK-MoE model

| Setting | Value |
| --- | --- |
| Batch size (Source Domain) | 4096 |
| Target mix ratio | 0.25 |
| Epochs | 100 |
| Optimizer | AdamW |
| Initial learning rate | 1×10^-4^ |
| Weight decay | 1×10^-2^ |
| LR scheduler | CosineAnnealingWarmRestarts (T_0_ = 10, T_mult_ = 2) |
| Early stopping patience | 5 epochs (no validation improvement) |
| Hidden dimension d_model_ | 64 |
| Number of layers | 4 |
| Gas experts | 2 |
| Concentration experts | 3 |
| Loss weights (w_zero_, w_gas_, w_conc_, w_aux_, w_div_) | (3.0, 2.0, 1.0, 1.0, 0.1) |

**Table S4.** Experimental setup and hyperparameter configurations for iTransformer and PatchTST models.

| Parameter | Configuration |
| --- | --- |
| d_model | 128 |
| n_heads | 4 |
| Layers | 3 |
| Patch_len | 20 for PatchTST |
| Stride | 10 for PatchTST |
| Dropout | 0.2 |
| Epochs | 100 |
| Batch_size | 256 |
| Lr | 5e^-4^ |
| Optimizer | AdamW |
| Weight_decay | 1e^-4^ |
| Input Window | 100 time steps |
| Sensor Channels | 4 (R_S1_, R_S2_, R_S3_, R_S4_) |
| Training Set | 10,965 samples  (from Source Domain) |
| Test Set | 2,742 samples  (from Source Domain) |
| External Validation Set | 1,523 samples  (from Target Domain) |

**5. Physics-Informed Feature Construction**

This section summarizes the physics-informed feature engineering implemented in the feature extraction scripts and documented in the project’s feature-design notes. The goal is to encode both magnitude and kinetic information from the MEMS array in a way that matches known reaction-transport physics while remaining compatible with tabular models.

**5.1 Layer 1: Magnitude and kinetic descriptors**

The first layer operates on log-ratio sensor responses and their time derivatives to preserve absolute signal strength and transient behaviour:

*Log-ratio response:* For each channel, the primary intensity feature is the log-ratio of the instantaneous resistance to a baseline reference,

$S\left( t \right)=\ln R\left( t \right)-\ln R_{\text{base}}(t)$	(S2)

which approximately linearizes the nonlinear conductance–concentration relationship and suppresses slow baseline drift.

*Signal velocity:* The first temporal derivative of the log-ratio signal,

$v(t)=\frac{dS(t)}{dt}$	(S3)

captures adsorption/desorption rates and highlights abrupt gas‑in and purge transitions.

*Kinetic hysteresis descriptor (KHD):* A hysteresis energy term

$E_{\text{hyst}}=\sinh^{-1}(\max(0,-S(t)v(t)))$	(S4)

is activated only when the signal is negative (adsorption phase) and the velocity points back toward equilibrium (recovery phase). This descriptor underpins the Zero‑Gate by distinguishing true Gas Absence from a recovering, still‑contaminated baseline.

*Phase angle and radius:* In the *phase space* spanned by ($S$, $\nu$), a phase angle

$\theta=\arctan\frac{kv}{S}$	(S5)

and a phase radius

$r=\sqrt{S^{2}+(kv)^{2}}$	(S6)

jointly encode the reaction stage (adsorption, quasi-equilibrium or desorption) and the combined potential/kinetic energy of the response.

*Trajectory roughness:* An exponentially weighted moving average of squared velocity,

$R_{\text{traj}}(t)=(1-\alpha)R_{\text{traj}}(t-1)+\alpha v(t)^{2}$	(S7)

quantifies signal noise and turbulence, aiding in the rejection of electronic noise spikes.

A total of 40 dynamic features are extracted from the linearized signal, including response amplitude, velocity, acceleration, and kinetic hysteresis descriptor, etc. The kinetic hysteresis descriptor quantifies the asymmetry between adsorption and desorption rates, providing robust descriptors for distinguishing gases that exhibit similar steady-state responses but follow different kinetic trajectories, thereby supporting stable concentration determination. These Layer 1 features retain amplitude information and are primarily consumed by concentration experts in the HK‑MoE.

**5.2 Layer 2: Shape, topology and thermofluid descriptors**

The second layer normalizes responses across channels to disentangle gas identity from absolute concentration:

*Normalized response vector:* Each multi‑channel snapshot yields a normalized vector $\hat{S}$ that preserves only the direction of the response pattern. This removes global gain changes while retaining the “shape” that characterizes gas identity.

*Normalized difference indices (NDI):* For each channel pair ($i$, $j$), a normalized difference index

$\text{NDI}_{ij}=\hat{S}_{i}-\hat{S}_{j}$	(S8)

amplifies relative contrasts between sensors and improves separation of chemically similar species.

*Direction cosines:* The components of $\hat{S}$ are treated as direction cosines,

$\cos\alpha_{i}=\hat{S}_{i}$	(S9)

providing an explicit geometric interpretation of the response pattern on a hypersphere.

*Response entropy and energy:* Interpreting the normalized magnitudes as a discrete distribution $p_{i}$, the Shannon entropy

$H=-\sum_{i} p_{i}\ln p_{i}$	(S10)

and the quadratic energy

$E=\sum_{i} p_{i}^{2}$	(S11)

jointly describe how concentrated or diffuse the array response is across channels.

*Thermal gradient and channel divergence:* A thermal slope

$\Delta_{\text{therm}}=\hat{S}_{\text{last}}-\hat{S}_{\text{first}}$	(S12)

encodes the effect of the imposed temperature gradient along the array, while a channel-divergence measure summarizes deviations of each channel from the mean response, providing a coarse fluid/flow signature.

A total of 13 morphological features are extracted, including direction cosines, pairwise normalized difference indices, and thermal-gradient descriptors. These features preserve the topological structure of gas responses under temperature modulation, ensuring that gas clusters remain separable even after drift, and thus enabling reliable gas classification.

Layer 2 features are fed mainly to gas-classification experts, offering shape and topology-based invariants that are robust to concentration changes and slow drift. Together, the two layers form a dual-stream representation that separates “how strong” the response is from “what pattern” it exhibits. **Table S5** summarizes the main physics-informed feature blocks used in the HK-MoE model.

**Table S5.** Grouping and description of key physics-guided and derived features used in the HK-MoE model.

| Feature Group | Count | Description | Typical Examples |
| --- | --- | --- | --- |
| Raw Resistance | 4 | Raw resistance from four sensing channels | Rs1, Rs2, Rs3, Rs4 |
| Log-Ratio | 4 | Natural log of resistance normalized by baseline | Rs1_log_ratio, … |
| Phase | 4 | Phase information from dynamic response | Rs1_phase_rad, … |
| NDI | 6 | Normalized difference index between channels | ndi_Rs1_Rs2, … |
| Derivatives | 8 | First and second order temporal derivatives | dRs1/dt, d2Rs1/dt2 |
| Thermodynamic | 2 | Slope and intercept from Arrhenius-like plots | therm_slope, therm_intercept |

**6. Evaluation Metrics**

To quantitatively assess model performance, this study adopts Precision, Recall, and the Macro-F1 score as the primary evaluation metrics.

*Precision*: Precision measures the proportion of true positives among all positive predictions, reflecting the model’s reliability to avoid false alarms, as defined in **Equation S13**:

$\text{Precision}_{i}=\frac{TP_{i}}{TP_{i}+FP_{i}}$	(S13)

where $TP_{i}$ and $FP_{i}$ denote the true positives and false positives of class $i$, respectively.

*Recall*: Recall measures the proportion of true positives correctly identified among all actual positive samples, indicating the model’s capability to detect target gases, as defined in **Equation S14**:

$\text{Recall}_{i}=\frac{TP_{i}}{TP_{i}+FN_{i}}$	(S14)

where $FN_{i}$ represents the false negatives of class $i$.

*Macro-F1 Score*: The F1-score is the harmonic mean of Precision and Recall, offering a balanced assessment of model performance. To comprehensively evaluate multi-class performance, this study reports the Macro-F1 score, which is the unweighted average of the F1-scores calculated for each class, as defined in **Equation S15**:

$\text{Macro-F1}=\frac{1}{C}\sum_{i=1}^{C} \frac{2\cdot\text{Precision}_{i}\cdot\text{Recall}_{i}}{\text{Precision}_{i}+\text{Recall}_{i}}$	(S15)

where $C$ is the number of classes. This metric treats all classes equally and prevents the majority “Background Air” class from dominating the overall evaluation.

**7. Ablation Configurations**

To validate the rationality of the model architecture and to quantify the contribution of each component to drift-robust performance, this study conducts a systematic ablation analysis along two key dimensions: (i) the hierarchical architectural components of the model and (ii) the physics-informed feature engineering pipeline. To quantify the overall performance change across all three tasks, we introduce Total Delta (Δ), calculated as the sum of percentage differences:

$Total \Delta=\Delta Pres\%+\Delta Class\%+\Delta Conc\%$	(S16)

$\Delta Pres\%=Pres.F1\left( Experiment \right)-Pres.F1(Ours)$	(S17)

$\Delta Class\%=Class.F1\left( Experiment \right)-Class.F1(Ours)$	(S18)

$\Delta Conc\%=Conc.F1\left( Experiment \right)-Conc.F1(Ours)$	(S19)

All F1-scores and Δ values are expressed as percentages, with positive Δ indicating performance improvement and negative Δ indicating degradation.

The performances of different model variants on the three tasks on the Target Domain are summarized in **Table S6**. The results show that both traditional baseline models (LSTM and LightGBM) and state-of-the-art Transformer-based time-series models (iTransformer and PatchTST) achieve substantially lower F1-scores for gas classification and concentration determination than the proposed HK-MoE model. These findings indicate that existing data-driven models exhibit limited robustness against sensor drift and the associated domain shift. In contrast, the proposed HK-MoE model leverages physics-guided feature decomposition and hierarchical expert routing to learn more transferable representations, thereby achieving superior cross-domain robustness.

**7.1 Impact of Architectural Components**

To assess the importance of the core structural modules, key components were selectively removed or replaced. Replacing the KAN experts [S1,S2] with a standard SwiGLU MLP [S3] (Exp-B) results in a slight performance drop (Total Delta = −1.36%), indicating that KAN layers better capture the nonlinear responses of MOS sensors, although their contribution is primarily reflected in fine-grained fitting accuracy. Removing the sparse expert-routing mechanism [S4,S5] (Exp-C) leads to a more pronounced degradation (Total Delta = −4.52%). This demonstrates that MoE routing enables different experts to specialize in specific gas types or concentration ranges, effectively decomposing complex drift patterns and improving model generalization.

The most substantial impact arises when the Zero-Gate component is removed (Exp-D). In this case, Pres. F1 drops to 88.17%, with a Total Delta of −28.68%, representing the largest decline among all architectural ablations. This confirms that Zero-Gate effectively suppresses false detections caused by baseline drift and serves as a critical mechanism for maintaining model stability on the Target Domain.

**7.2 Necessity of Physics-Informed Features**

To evaluate the contribution of each feature group, the physics-informed features were removed category by category. Among them, the statistical features (Exp-F2) and the kinetic-phase features (Exp-F5) contribute most significantly to model robustness. Statistical features provide fundamental distribution descriptors that help distinguish signal from noise; removing them (Exp-F2) reduces the Class. F1 to 96.48% (Total Delta = −9.68%). Removing the kinetic-phase features (Exp-F5) leads to a similar decline (Total Delta = −15.41%), highlighting the importance of capturing the dynamic response trajectories of the sensors (e.g., adsorption/desorption rates), which remain relatively stable even under baseline drift.

Eliminating all physics-informed features (Exp-F6) and relying solely on the raw four-channel resistance signals results in a catastrophic performance drop. In this setting, Pres. F1 decreases to 89.92%, Class. F1 to 31.13%, and Conc. F1 to 26.86%, with a Total Delta of −87.95%. These results indicate that the raw resistance signals lack the domain-invariant properties required for reliable gas recognition.

Overall, the findings demonstrate that the superior performance of HK-MoE does not arise from any single component. Instead, it emerges from the deep synergy between domain-invariant physical features, the hierarchical sparse-expert architecture, and the Zero-Gate physical constraint.

**Table S6.** Ablation study on Target Domain F1-scores (external validation set).

| Exp ID | Variant Description | Pres. F1 | Class. F1 | Conc. F1 | Total Delta |
| --- | --- | --- | --- | --- | --- |
| **Ours** | **Full HK-MoE Model (KAN + DA)** | **97.55%** | **80.62%** | **57.68%** | **0.00%** |
| iTransformer | Inverted Transformer | 98.99% | 30.19% | 44.57% | -62.10% |
| PatchTST | Patch-wise Transformer | 96.36% | 19.09% | 35.02% | -85.38% |
| Baseline-LSTM | LSTM Baseline (Sequential, Multi-task) | 98.85% | 35.50% | 43.54% | −57.96% |
| Baseline-GBM | LightGBM Baseline (Tabular, Multi-task) | 98.49% | 25.63% | 51.92% | −59.81% |
| Exp-B | No KAN Expert  (SwiGLU MLP) | 97.26% | 80.10% | 57.13% | −1.36% |
| Exp-C | No MoE  (Dense KAN Encoder) | 97.72% | 81.21% | 52.40% | −4.52% |
| Exp-D | No Zero-Gate  (Air Detection) | 88.17% | 71.95% | 47.05% | −28.68% |
| Exp-F1 | No G1 Raw Response Features | 96.23% | 76.61% | 55.43% | −7.58% |
| Exp-F2 | No G2 Statistical Features | 96.48% | 75.67% | 54.03% | −9.68% |
| Exp-F3 | No G3 Physical Pattern Features | 96.56% | 77.10% | 56.03% | −6.16% |
| Exp-F4 | No G5 Temporal Velocity Features | 96.88% | 78.91% | 58.86% | −1.20% |
| Exp-F5 | No G6 Kinetic Phase Features | 95.95% | 75.11% | 49.39% | −15.41% |
| Exp-F6 | 4-Channel Raw Only  (No Feature Engineering) | 89.92% | 31.13% | 26.86% | −87.95% |
| Exp-G | No Temporal + Kinetic Features | 93.94% | 78.58% | 44.11% | −19.22% |

**8. Few-Shot Calibration for Drift Recovery**

Although domain-adaptation methods can align the source and target distributions at a global level, real deployment environments often exhibit more complex local drift patterns that require additional lightweight calibration. In this study, a few-shot calibration scheme is adopted, where the HK-MoE model is fine-tuned using only a small number of labeled Target-Domain samples (*k*-shots). This enables rapid performance recovery under drift conditions with minimal annotation cost. Because drift affects different concentration ranges unevenly, a concentration-balanced sampling strategy is used to construct the support set. This ensures coverage of the full dynamic concentration range and improves both calibration efficiency and stability.

**Figure S13**(a) compares HK-MoE with Maximum Mean Discrepancy (MMD) [S6], Correlation Alignment (CORAL) [S7], and their combination (MMD + CORAL) on the Target Domain across the three tasks. HK-MoE achieves the highest F1-scores for gas presence detection, gas classification, and concentration determination, demonstrating that the structural design combining physics-informed features, hierarchical MoE, and the Zero-Gate constraint is substantially more effective than traditional distribution-alignment methods when handling complex drift. Figure S13(b) shows the performance recovery of the full HK-MoE and its structural variants (without KAN, without MoE, without Zero-Gate) under a linear-probe setting with only 10 labeled samples (*k* = 10). After few-shot fine-tuning, the complete HK-MoE achieves significant improvements across all tasks (Pres. F1 = 98.61%, Class. F1 = 79.70%, Conc. F1 = 72.00%). In contrast, removing KAN or MoE noticeably weakens the recovery (w/o KAN: Pres. F1 = 96.26%, Class. F1 = 84.20%, Conc. F1 = 67.39%; w/o MoE: Pres. F1 = 98.63%, Class. F1 = 83.53%, Conc. F1 = 72.07%), while removing Zero-Gate leads to severe degradation across all tasks (Pres. F1 = 34.36%, Class. F1 = 7.476%, Conc. F1 = 36.13%). These results indicate that KAN experts are essential for modeling nonlinear drift, MoE routing supports task specialization and residual correction, and Zero-Gate provides critical baseline-stability constraints. Figure S13(c) and (d) present the F1 trends under different *k* values (0, 5, 10, 20, 50, 100) for gas-balanced and concentration-balanced sampling strategies. The concentration-balanced strategy consistently yields faster convergence and lower variance across all *k* values, achieving near-complete recovery even at *k* = 10. This demonstrates that when drift varies across concentration ranges, concentration-balanced sampling more effectively captures the underlying drift patterns, thereby substantially improving the efficiency and stability of few-shot calibration.

To further evaluate the long-term stability and drift performance of the proposed system, the device previously aged for 30 days was subjected to an additional 30 days of continuous aging under the same operating conditions. The 60-day aging dataset (20260715_Combined_SnO2_test_data.csv) exhibits significantly more severe drift with baseline resistance shifts exceeding 40% in some channels. Without calibration, performance degrades significantly: Pres. F1 drops to 94.83%, Class. F1 to 53.59%, and Conc. F1 to 30.65%. These results indicate long-term drift fundamentally alters sensor response characteristics beyond pre-trained domain-adaptation capabilities. With only 100 labeled samples (*k* = 100) using the concentration-balanced strategy, the proposed calibration achieves significant recovery: Pres. F1 improves to 94.71%, Class. F1 to 83.50%, and Conc. F1 to 72.92%. These results demonstrate that concentration-balanced few-shot calibration effectively captures complex drift patterns accumulated over 60 days, enabling rapid performance recovery even under extreme conditions. **Figure S14** illustrates performance comparison across 30-day and 60-day drift conditions, confirming consistent recovery efficiency, with detailed F1-scores without and with few-shot calibration are summarized in **Table S7**.

**Figure S13.** Few-shot calibration and domain adaptation dynamics. (a) Comparative benchmark of Proposed HK-MoE against standard baselines on the Target Domain. (b) Detailed breakdown of architectural component contributions under few-shot fine-tuning. Performance recovery curves as a function of Target Domain support shots for (c) gas-balanced and (d) concentration-balanced sampling strategies.

**Figure S14.** Drift compensation and *k*-shot (*k* = 100) domain adaptation performance across the three tasks. Performance comparison between HK-MoE and *k*-shot adapted HK-MoE across Source Domain, Target Domain (30-day aging), and 60-day aging conditions for (a) target gas presence detection, (b) gas classification, and (c) concentration determination.

**Table S7.** Comparison of F1-scores without and with few-shot calibration.

|  | HK-MoE | | | *k*-shot adapted HK-MoE (*k* = 100) | | |
| --- | --- | --- | --- | --- | --- | --- |
| Dataset | Pres. F1 | Class. F1 | Conc. F1 | Pres. F1 | Class. F1 | Conc. F1 |
| Target Domain  (30-day aging) | 97.55% | 80.62% | 57.68% | 98.99% | 89.07% | 88.49% |
| 60-day aging | 94.83% | 53.59% | 30.65% | 94.71% | 83.50% | 72.92% |

**9. Mixed-Gas Operation Results**

To address practical multi-gas monitoring scenarios, we developed a multi-label interval classification framework for simultaneous identification of gas composition and concentration levels in mixed-gas environments. The framework extends the HK-MoE architecture with a CORAL (Correlation Alignment) layer to handle complex multi-gas interactions. The dataset consists of four-gas mixtures (H_2_, CO, CH_4_, CO_2_) at varying concentrations (100, 300, 500 ppm), with 80%/20% stratified train/test split by concentration tuples. Feature engineering is performed through physics-guided feature extraction, capturing transient response characteristics and steady-state properties across multiple thermal cycles.

**Table S8.** Task-wise F1-scores for multi-component gas samples.

| Gas mixtures | Pres. F1 | Class. F1 | Conc. F1 |
| --- | --- | --- | --- |
| Mix 1 | 99.86% | 99.79% | 99.86% |
| Mix 2 | 99.96% | 99.93% | 99.90% |
| Mix 3 | 99.95% | 99.93% | 99.95% |
| Mix 4 | 99.91% | 99.86% | 99.62% |
| Overall | 99.92% | 99.88% | 99.83% |

**Table S9.** F1-scores for each characteristic gas using the complete multi-component gas dataset.

| Gas | Class. F1 | Conc. F1 |
| --- | --- | --- |
| H_2_ | 99.75% | 99.86% |
| CO | 99.86% | 99.86% |
| CH_4_ | 99.93% | 99.86% |
| CO_2_ | 99.72% | 99.83% |

**Table S10.** Comparison of other state-of-the-art gas-sensing systems dedicated to battery monitoring.

| Sensor | Target  Gases | Model | Single Gas Discrimination | Mixed-Gas  Deconvolution | Long-Term  Stability | Ref. |
| --- | --- | --- | --- | --- | --- | --- |
| Eight commercial MOS sensors | H_2_, CO, and C_2_H_4_ | SACNN‑Mamba | / | Conc. prediction:  R^2^ (H_2_) = 99.74 %;  R^2^ (CO) =99.45 %;  R^2^ (C_2_H_4_) =99.48 % | / | [S8] |
| Three commercial MOS sensors | H_2_, CO, and their mixes | Multilayer perceptron (MLP) | Class. accuracy = 99.23% | Conc. prediction:  RMSE (H_2_) = 0.0537;  RMSE (CO) = 0.1056,  RMSE (mixes) = 0.0956 | / | [S9] |
| Three commercial MOS sensors | H_2_, CO, and their mixes | ISA-LSTM-TCN | Class. accuracy = 99.23% | Conc. prediction:  R^2^ (H_2_) = 0.9510;  R^2^ (CO) = 0.8400 | / | [S10] |
| Two self-made and three commercial MEMS MOS sensors | H_2_, C_2_H_4_, C_2_H_2_, CH_4_, CO, and Ethanol | A four-layer MLP architecture | Class. accuracy = 98.5% | / | / | [S11] |
| **A self-made 2×2 single-chip MEMS sensor**  **array** | **H_2_, CO, CH_4_, and CO_2_** | **The HK-MoE model** | **Pres. F1 = 99.53%;**  **Class. F1 = 99.88%;**  **Conc. F1 = 98.09%** | **Conc. prediction:**  **F1 (H_2_) = 99.86%;**  **F1 (CO) = 99.86%;**  **F1 (CH_4_) =99.86%;**  **F1 (CO_2_) =99.83%** | **60-day aging with few-shot calibration:**  **Pres. F1 = 94.71%;**  **Class. F1 = 83.50%;**  **Conc. F1 = 72.92%** | **This work** |

**References**

[S1]	Z. Liu, Y. Wang, S. Vaidya, F. Ruehle, J. Halverson, M. Soljačić, T. Y. Hou, and M. Tegmark, “KAN: Kolmogorov-Arnold networks,” *arXiv:2404.19756* (2024). https://doi.org/10.48550/arXiv.2404.19756

[S2]	Z. Li, “Kolmogorov-Arnold networks are radial basis function networks,” *arXiv:2405.06721* (2024). https://doi.org/10.48550/arXiv.2405.06721

[S3]	R. A. Fisher, “Statistical methods for research workers,” in *Breakthroughs in Statistics: Methodology and Distribution* (Eds: S. Kotz, N. L. Johnson), Springer, 1992, 66–70. https://doi.org/10.1007/978-1-4612-4380-9_6

[S4]	R. A. Jacobs, M. I. Jordan, S. J. Nowlan, and G. E. Hinton, “Adaptive mixtures of local experts,” *Neural Computation* 3 (1991): 79. https://doi.org/10.1162/neco.1991.3.1.79

[S5]	N. Shazeer, A. Mirhoseini, K. Maziarz, A. Davis, Q. Le, G. Hinton, and J. Dean, “Outrageously large neural networks: the sparsely-gated mixture-of-experts layer,” *arXiv:1701.06538* (2017). https://doi.org/10.48550/arXiv.1701.06538

[S6]	A. Gretton, K. M. Borgwardt, M. J. Rasch, B. Schölkopf, and A. Smola, “A kernel two-sample test,” *Journal of Machine Learning Research* 13 (2012): 723. https://jmlr.org/papers/v13/gretton12a.html

[S7]	B. Sun and K. Saenko, “Deep CORAL: correlation alignment for deep domain adaptation,” in *Computer Vision – ECCV 2016 Workshops* (Eds: G. Hua, H. Jégou), Springer, 2016, 443–450. https://doi.org/10.1007/978-3-319-49409-8_35

[S8]	M. Tang, X. Zhang, C. Zhang, et al., “An Intelligent Sensing Array for Thermal Runaway Characteristic Gas Concentration Prediction Based on SACNN-Mamba,” *Sensors and Actuators B: Chemical* *431* (2025): 137368, https://doi.org/10.1016/j.snb.2025.137368.

[S9]	Pu, M. Yang, M. Jiao, D. Zhao, Y. Huo, and Z. Wang, “Thermal Runaway Warning of Lithium Battery Based on Electronic Nose and Machine Learning Algorithms,” *Batteries* *10*, no. 11 (2024): 390, https://doi.org/10.3390/batteries10110390.

[S10]	R. Guo, Q. Yu, H. Li, Z. Pu, and M. Jiao, “Rapid Detection of Mixed Gases from Lithium Battery Thermal Runaway Based on ISA-LSTM-TCN,” *Batteries* *12*, no. 6 (2026): 188, https://doi.org/10.3390/batteries12060188.

[S11]	H. Liu, S. Zhang, S. Xue, et al., “Design of MEMS Gas Sensors and Integration for Multiple Gas Classification for Lithium-Ion Battery Thermal Runaway Warning,” *Materials* *19*, no. 11 (2026): 2419, https://doi.org/10.3390/ma19112419.
